# Supplementary material for: Novel Coronavirus Infection (COVID-19) in Humans: A Scoping Review and Meta-Analysis
Source: J Clin Med. 2020 Mar 30;9(4):941. doi: 10.3390/jcm9040941 (PMC7230636; doi:10.3390/jcm9040941)
Supplement: Supplementary file 1 [file jcm-09-00941-s001.pdf]

Novel coronavirus (SARS-CoV-19) infection in humans: a scoping review and meta-analysis

Tables

The following pages are destinated for all tables mentioned in the full text

Table S1. Main characteristics of included studies.

Study	Recruitment	Scenario	Participants (n)	Mean age in years (age-range)	Male/Female-ratio
Australian Government Department of Health (B) [32]	All reports until February 15, 2020	Patients admitted to different hospitals in Australia	15	43 (8–66)	1.5/1
Bai, S. et al (B) [33]	NA	Patients admitted at the ED in Gansu	7	NA	NA
Bai, Y. et al (B) [34]	January 2020	Patient admitted to the Fifth People’s Hospital of Anyang	6	NA (42–57)	0.2/1
Bastola, A. et al (A) [35]	NA	Patient admitted to the Sukraraj Tropical and Infectious Disease Hospital,	1	30(NA)	NA
Bernheim, A. et al (B) [25]	January 18 - February 2	Patients admitted to 4 different hospitals from 4 different provinces in China	121	45.3 (18–80)	1.01
Cai, J. et al (B) [36]	NA	Patient admitted at the Children's Hospital of Fudan University	1	7(NA)	NA

Chan, J. et al (B) [6]	NA	Patients admitted to The University of Hong Kong-Shenzhen Hospital, Shenzhen	6	46 (10–66)	1/1
Chang, L. et al (B) [37]	January 16 - 29	Patients admitted to hospitals in Beijing (Beijing Tsinghua Changgung Hospital, Beijing Anzhen Hospital, Chinese PLA General Hospital)	13	34 (2–NA)	3.34/1
Chen, F. et al (A) [38]	NA	Patient admitted to the ED of the Wuhan Children's Hospital	1	13 months	1/0
Chen, L. et al (B) [16]	January 14 - 29	Patients admitted at the Tongji hospital	29	NA (26–79)	2.62/1
Chen, N. et al (B) [39]	January 1 - 20	Patients admitted at the Jinyintan Hospital in Wuhan	99	55.5 (21-88)	3.09/1
Chung, M. et al (B) [40]	January 18 - 27	Patients admitted to Zhuhai, Nanchang and Qingdao Hospital	21	- 51 (29-77)	1.62/1
Duan, Y. et al (A) [41]	NA	Patient admitted to the Third Affiliated Hospital of Sun	1	46	0/1

		Yat-sen University			
Fang, X. et al (A) [42]	NA	Patient admitted to the ED of the West China Hospital	1	47	1/0
Fang, Y. et al (B) [43]	January 19 - February 4	Patients admitted to the Taizhou Enze Medical Center	51	45 (NA)	1.31/1
Fang Y. et al (B) [44]	NA	Patients admitted to Affiliated Taizhou Hospital of Wenzhou Medical University	2	38.5 (32-45)	1/1
Feng K. et al (B) [17]	January16 to February 6	Patient admitted at the Shenzhen Third People's Hospital	15	7 (4-14)	0.5/1
Giovanetti, M. et al (B) [45]	NA	Patient admitted to the Rome's Spallanzani Hospital	2	NA	NA
Han, W. et al (A) [92]	NA	Patient admitted to the People's Hospital in Wuwei	1	47 (NA)	1/0
Hao, W. et al (A) [46]	NA	Patient admitted to the Affiliated Hospital of Yan'an University	1	60 (NA)	1/0
Holshue, M. et al (A) [47]	NA	Patient admitted at the Urgent care clinic in Snohomish County, Washington	1	35 (NA)	1/0

Hu, J. et al (C) [20]	All reports until February 14, 2020	Epidemiological report evaluating the exported risk of novel coronavirus pneumonia across China	49,970	NA	NA
Huang, C. et al (B) [48]	All reports until January 2, 2020	Patients admitted to a designated hospital in Wuhan	41	49 (NA)	2.70/1
Huang P. et al (A) [49]	NA	Patient admitted to the Guangdong Hospital of Traditional Chinese Medicine	1	36 (NA)	1/0
Jie, L. et al (B) [94]	January 22 - February 11	Patients admitted to the local hospital in Dazhou	17	45 (22-65)	1.43/1
Kaiyuan, S. et al (C) [22]	January 20-31	Patients data were associated to crowdsourced reports from DXY.cn	507	46 (35-60)	1.22/1
Ki M. et al (B) [51]	All reports until February 8, 2020	Patients admitted to local hospitals in Korea	24	42(21-62)	1.4/1
Lei J. et al (A) [52]	NA	Patients admitted to The First Hospital of Lanzhou University	1	33	0/1
Li Q. et al (B) [53]	All reports until January 22, 2020	Patients admitted in different locations in China	425	59 (15-89)	1.27/1

Lin X. et al (B) [54]	NA	Patients admitted to Jiangxi Provincial People's Hospital	2	37 (35-39)	2/0
Liu C. et al (B) [55]	January 23 - February 8	Patients were admitted to 7 different hospitals	32	38.5 (26.25-45.75)	1.66/1
Liu K. et al (B) [56]	December 30 - January 24	Patients admitted in nine tertiary hospitals in Hubei province	137	55 (20-83)	0.8/1
Liu M. et al (B) [57]	January 10 - 31	Patients admitted at the Jianghan University Hospital	30	35 (21-59)	0.5/1
Liu P. et al (A) [58]	NA	Patient admitted in Wuhan	1	41	0/1
Ministry of Health, Labour and Welfare, Japan (A) [59]	NA	Patient admitted to local ED in Japan	1	NA	1/0
Ministry of Public Health (MoPH), Thailand (A) [60]	NA	Patient admitted to local ED in Thailand	1	61	0/1
Pan F et al (B) [61]	January 12 - February 6	Patient admitted to the Union Hospital	21	40 (25-63)	0.4/1
Pan, Y. et al (B) [19]	December 30 – January 31	Patients admitted at the ED of the Tongji Hospital	63	44.9 (NA)	1.1/1
Phan L et al (B) [62]	January 2	Patients admitted to Cho Ray Hospital	2	46 (27-65)	2/0

Pongpirul, W. et al (A) [63]	NA	Thai taxi driver admitted at the primary care clinic in Bangkok	1	51	1/0
Ren L et al (B) [64]	December 18 - 29	Patients admitted to the ED of the Jin Yin-tan Hospital of Wuhan	5	53.6 (41-65)	1.5/1
Rothe C et al (A) [65]	NA	Patient admitted to the University Hospital LMU Munich	1	33	1/0
Shi H et al (A) [66]	NA	Patient admitted to the Union Hospital, Wuhan	1	42	1/0
Silverstein, W. et al (A) [67]	NA	Patient admitted to the Emergency Department in Toronto	1	56	1/0
Song, F. et al (B) [68]	January 20 - 27	Patients admitted to the Shanghai Public Health Clinical Center	51	NA (16-76)	0.96/1
Tang N. et al (B) [18]	NA	Patients admitted at the ED of the Tongji Hospital of Huazhong University of Science and Technology	183	54.1 (14-94)	1.15/1
Wang W. (B) [69]	December 1 - January 26	Patients registered in Chinese Governmental Databases	1975	75 (48-89)	NA

Wei M. et al (B) [70]	December 8 - February 6	Patients (pediatric) admitted in any hospital in China	9	NA (1 month 26 days - 11 months)	0.28/1
Wei-ji, G. et al (B) [71]	All reports until January 29, 2020	Patients admitted to 552 hospitals in 31 provinces	1099	47 (NA)	1.39/1
Xingzhi, X. et al (B) [72]	NA	Patients obtained in a database (Radiology Quality Control Center, Hunan Province)	5	48 (25-66)	4/1
Xu X. et al. (A) [73]	NA	Patient previously healthy admitted to the ED in the First Affiliated Hospital, Jinan University	1	53 (NA)	1/0
Xu X. et al (B) [74]	January 10 - January 26	Patients admitted to different hospitals in Zhejiang province	62	41 (NA)	1.81/1
Yang Y. et al. (C) [90]	All reports until January 26, 2020	Patients admitted to 30 different provinces in China	4021	49 (NA)	1.22/1
Yingxia, L. et al. (B) [23]	NA	Patients admitted at the Shenzhen Third People's Hospital	12	62.5 (10-72)	2/1
Zeng L. et al. (A) [75]	NA	Patient admitted to the ED of the Wuhan Children's Hospital	1	17 days	1/0

Zhang J. et al. (B) [76]	January 16 - February 3	Patients admitted to the Zhongnan Hospital of Wuhan University	140	57 (25-87)	1.02/1
Zhang M. et al. (B) [77]	January 18 - February 3	Patients admitted at the Beijing Tsinghua Changgeng Hospital	9	36 (15-49)	1.25/1
Zhang Y. et al. (A) [78]	NA	Patient admitted to the ED in Xiaogan	1	3months	0/1
Zhang Z. et al. (B) [79]	NA	Patients admitted at the Renmin Hospital of Wuhan University	2	38 (NA)	1/1
Zhu, N. et al (B) [91]	NA	Patient admitted at Wuhan Hospital	3	47.3 (32-61)	2/1

Legend: (A) Case Reports. (B) Case Series (C) Epidemiological Reports

Table S2. Main characteristics of excluded studies (mainly due to patients overlapping).

Study	Recruitment	Scenario	Participants (n)	Mean age in years (age-range)	Male/Female-ratio
Park et al, 2020 [80]	NA	Patient admitted to a specific hospital in Korea	1	35	NA
Lim et al, 2020 [81]	January 26	Patient admitted to a public health center at Myongji Hospital	1	54	NA
Kim et al, 2020 [82]	January 18 - January 31	Patient admitted to a local clinic in Wuhan	1	35	NA
Yoo et al, 2020 [83]	February 07	Patients confirmed by the Korea Center for Disease Control and Prevention	24	21 - 62	1.4/1
Kim et al, 2020 [84]	January 19 - February 06	Patients from Wuhan, China quarantined at the airport in Korea	2	45 (35 – 55)	1/1
COVID-19 National Incident Room Surveillance Team, 2020 [85]	January 26 - February 01	Patients reported a travel history to China, and 92% (11/12) had a travel history to Wuhan, Hubei Province, China	12	45 (21–66)	1.4/1
COVID-19 National Incident Room Surveillance Team, 2020 [86]	Week ending 08 February	Patients reported a travel history to China, and 80% (12/15) had a travel history to Wuhan, Hubei Province, China	15	43 (8–66)	1.5/1

Wang et al, 2020 [93]	January 01 - January 28	Patients with confirmed at Zhongnan Hospital of Wuhan University in Wuhan, China	138	NA (22-92)	1.2/1
Wang et al, 2020 [87]	January 21 - January 24	Patients admitted to Shanghai Public Health Clinical Center	4	44 (19 -63)	3/1
Chen et al, 2020 [50]	January 20 - January 31	Pregnant patients admitted to Zhongnan Hospital of Wuhan University, Wuhan, China	9	NA (26 – 40)	0/9
Chen et al, 2020 [88]	December 25 - January 12	Patients admitted to Zhongnan Hospital of Wuhan University	2	30 (21 -39)	1/1
Bajema et al, 2020 [89]	January 17 - January 31	Patients reported a travel history to China and patients reported close contact with a person being evaluated for SARS-CoV-19 infection	210	NA (21–49)	2.8/1
Epidemiological group of emergency response mechanism of new coronavirus pneumonia in Chinese CDC (C) [21]	All reports until February 11, 2020	Epidemiological report evaluating the exported risk of novel coronavirus pneumonia across China	44672	NA (0-99)	1.06/1

Table S3. Risk of bias assessment.

	Selection	Ascertainment	Causality	Causality	Causality	Reporting
Study	1. Does the patient(s) represent(s) the whole experience of the investigator (center) or is the selection method unclear to the extent that other patients with similar presentation may not have been reported?	2. Was the exposure adequately ascertained?	3. Was the outcome adequately ascertained?	4. Were other alternative causes that may explain the observation ruled out?	7. Was follow-up long enough for outcomes to occur?	8. Is the case(s) described with sufficient details to allow other investigators to replicate the research or to allow practitioners make inferences related to their own practice?
Zhang, Y. et al	1	1	1	1	1	1
Chen, L. et al	1	1	1	1	1	1
Feng, K. et al	1	1	NA	1	NA	1
Bai, S. et al	1	1	1	1	1	1
Zhang, M. et al	1	1	1	1	1	1
Cai, J. et al	1	1	1	1	1	1
Zeng, L. et al	1	1	1	1	1	1
Chen, F. et al	1	0	1	1	1	1
Liu, C. et al	1	1	0	1	0	1
Hu, J. et al*	NA	NA	NA	NA	NA	NA
Liu, P. et al	0	1	0	1	0	0
Chan, J. et al	1	1	1	1	1	1
Zhu, N. et al	1	1	1	1	1	1
Tang, N. et al	1	1	1	1	1	1
Fang, X. et al	0	1	NA	1	NA	1
Bernheim, A. et al	1	1	NA	1	NA	1
Xingzhi, X. et al	1	1	0	1	0	1

Yingxia, L. et al	1	1	1	1	1	1
Zhang, J.et al	1	1	0	1	0	1
Guan W. et al	1	1	1	1	1	1
Liu M. et al	1	1	1	1	1	1
Liu K., et al	1	1	1	1	1	1
Zhang, Z. et al.	1	1	1	1	1	1
Huang, C. et al	1	1	1	1	1	1
Xu, X. et al	1	1	1	1	1	1
Australian Goverment Department of health	1	0	1	1	1	1
Chung, A. et al	1	1	NA	1	NA	1
Lei, J. et al	0	1	0	1	0	0
Fang, Y. et al.	0	1	1	1	1	1
Kaiyuan, S. et al*	NA	NA	NA	NA	NA	NA
Li, Q. et al	1	1	0	1	0	1
Song, F. et al.	1	1	NA	1	NA	1
Chang, L. et al	1	1	1	1	1	1
Ki, M. et al*	NA	NA	NA	NA	NA	NA
Jie, L. et al	1	1	1	1	1	1
Chen, N. et al	1	1	1	1	1	1
Yang, Y. et al	1	1	1	1	1	0
Shi, H. et al	0	0	NA	1	NA	1
Hao, W. et al	1	1	1	1	1	1
Holshue, M. et al	1	1	1	1	1	1
Silverstein, W. et al	1	1	1	1	1	1
Ren, L. et al	0	1	1	1	1	1
Xu, X. et al	0	1	0	1	0	1
Phan, L. et al	1	1	1	1	1	1
Pongpirul, W. et al	1	1	1	1	1	1

Ministry of Health, Labour and Welfare, Japan	1	1	1	1	1	1
Ministry of Public Health (MoPH), Thailand	1	1	1	1	1	1
Wei, M. et al	1	1	1	1	1	1
Lin, X. et al	0	1	NA	1	NA	1
Duan, Y. et al	0	1	1	1	1	1
Bai, Y. et al	1	1	1	1	1	1
Fang, Y. et al	1	1	NA	1	NA	1
Han, W. et al	0	1	1	1	1	1
Bastola, A. et al	1	1	1	1	1	1
Giovanetti, M. et al	1	1	0	1	0	0
Pan, F. et al	1	1	1	1	1	1
Pan, Y. et al	0	1	NA	1	NA	1
Rothe, C. et al	1	1	1	1	1	1
Wang, W. et al *	NA	NA	NA	NA	1	1
Huang, P. et al	0	0	NA	1	0	1

Legend: 0 = No; 1 = Yes; NA = Not applicable. * The risk of bias tool is not applicable for these studies.Domains associated to casualty (“Was there a challenge/rechallenge phenomenon?” And “Was there a dose–response effect?”) were removed because of no applicability to our study question.

Novel coronavirus (SARS-CoV-19) infection in humans: a scoping review and meta-analysis

Supplementary appendix

The following pages are destinated for all supplemental appendix mentioned in the full text

Supplemental material S1. PROSPERO registration copy

Systematic review

This record cannot be edited because it is being assessed by the editorial team

1. * Review title.

Give the working title of the review, for example the one used for obtaining funding. Ideally the title should state succinctly the interventions or exposures being reviewed and the associated health or social problems. Where appropriate, the title should use the PI(E)COS structure to contain information on the Participants, Intervention (or Exposure) and Comparison groups, the Outcomes to be measured and Study designs to be included.

The novel 2019 coronavirus (nCoV) infection in humans: A Systematic Review Protocol

2. Original language title.

For reviews in languages other than English, this field should be used to enter the title in the language of the review. This will be displayed together with the English language title.

Not applicable

3. * Anticipated or actual start date.

Give the date when the systematic review commenced, or is expected to commence.

19/02/2020

4. * Anticipated completion date.

Give the date by which the review is expected to be completed.

16/04/2020

5. * Stage of review at time of this submission.

Indicate the stage of progress of the review by ticking the relevant Started and Completed boxes. Additional information may be added in the free text box provided.

Please note: Reviews that have progressed beyond the point of completing data extraction at the time of initial registration are not eligible for inclusion in PROSPERO. Should evidence of incorrect status and/or completion date being supplied at the time of submission come to light, the content of the PROSPERO record will be removed leaving only the title and named contact details and a statement that inaccuracies in the stage of the review date had been identified.

This field should be updated when any amendments are made to a published record and on completion and publication of the review. If this field was pre-populated from the initial screening questions then you are not able to edit it until the record is published.

The review has not yet started: No

Review stage	Started	Completed

Supplemental material S2. Search strategy: "Novel coronavirus infection in humans: a scoping review and meta-analysis"

PubMed, Embase (Elsevier), LILACS, Scopus (Elsevier) and Cochrane CENTRAL were included in the search strategy. The searches were performed from January 1 2020 to February 24, 2020. Investigator/information specialist: Maria Björklund (Cochrane Sweden, Lund University, Sweden)

PubMed

(((((coronavirus[MeSH Terms]) OR coronavirus infections[MeSH Terms]) OR "betacoronavirus"[MeSH Terms]) OR "betacoronavirus 1"[MeSH Terms]) OR (Coronaviruses OR "Coronavirus Infection" OR "COVID-19" OR "Coronavirus Infection Disease 2019" OR "2019 Novel Coronavirus Infection" OR "2019-nCoV Infection" OR "2019 nCoV Infection" OR "2019-nCoV Infections" OR Betacoronavirus* OR "Novel Coronavirus Pneumonia" OR "2019 novel coronavirus" OR "coronavirus disease 2019" OR "nCoV" OR covid* OR "bat coronavirus"))

Limit: From 2019/01/01 to 2020/02/24

1380 records

Embase (Elsevier)

'coronavirinae'/exp OR 'betacoronavirus'/exp OR 'betacoronavirus 1'/exp OR coronaviruses OR 'coronavirus infection'/exp OR 'coronavirus infection' OR 'covid-

19' OR 'coronavirus infection disease 2019' OR '2019 novel coronavirus infection' OR '2019-ncov infection' OR '2019 ncov infection' OR '2019-ncov infections' OR betacoronavirus* OR 'novel coronavirus pneumonia' OR '2019 novel coronavirus'/exp OR '2019 novel coronavirus' OR 'coronavirus disease 2019' OR 'ncov' OR covid*

AND [embase]/lim NOT ([embase]/lim AND [medline]/lim

Limit: From 2019 to 2020/02/24

501 records

Latin American and Caribbean Center on Health Sciences Information (LILACS)

MH:("Coronavirus Infection") OR ("Infecciones por Coronavirus") OR ("Infecções por Coronavirus") OR ("Coronavirus Infection") OR ("Infection, Coronavirus") OR ("Infections, Coronavirus") OR "Coronavirus" OR "Coronavirus" OR "Coronavirus" OR MH:C02.782.600.550.200$ OR MH:B04.820.504.540.150$

Limit: Publication date 2019-2020

23 records

Scopus (Elsevier)

("coronavirus" OR "coronavirus infections" OR "betacoronavirus" OR "betacoronavirus 1" OR "coronaviruses" OR "Coronavirus Infection" OR "COVID-19" OR "Coronavirus Infection Disease 2019" OR "2019 Novel Coronavirus Infection" OR "2019-nCoV infection" OR "2019 nCoV Infection" OR "2019-nCoVinfections" OR "betacoronavirus*"

OR "Novel Coronavirus Pneumonia" OR "2019 novel coronavirus" OR "coronavirus disease 2019" OR "nCoV" OR "covid" OR "coronavirinae")

Limit: From 2019 to 2020/02/24 AND NOT INDEX(medline)

773 records

Cochrane CENTRAL

(MeSH descriptor: [Coronavirus] explode all trees OR MeSH descriptor: [Betacoronavirus] explode all trees OR MeSH descriptor: [Coronavirus Infections] explode all trees OR Coronavirus* OR betacoronavirus* OR nCoV* OR novel coronavirus* OR novel corona virus OR covid*)

Limit: Publication date 20190101-20200224

24 records

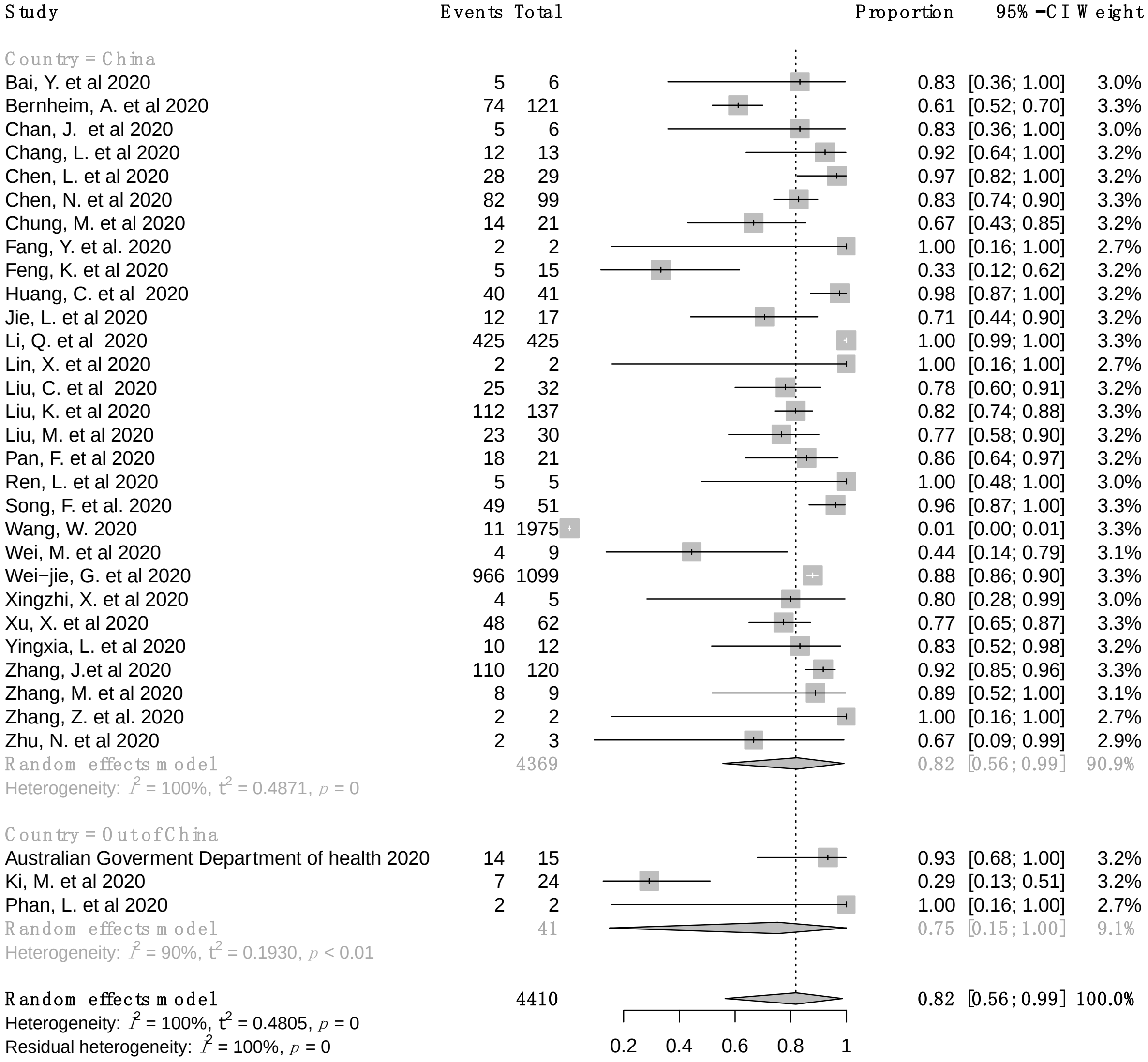

Figure S1. Meta-analysis of the incidence of fever among the selected studies.

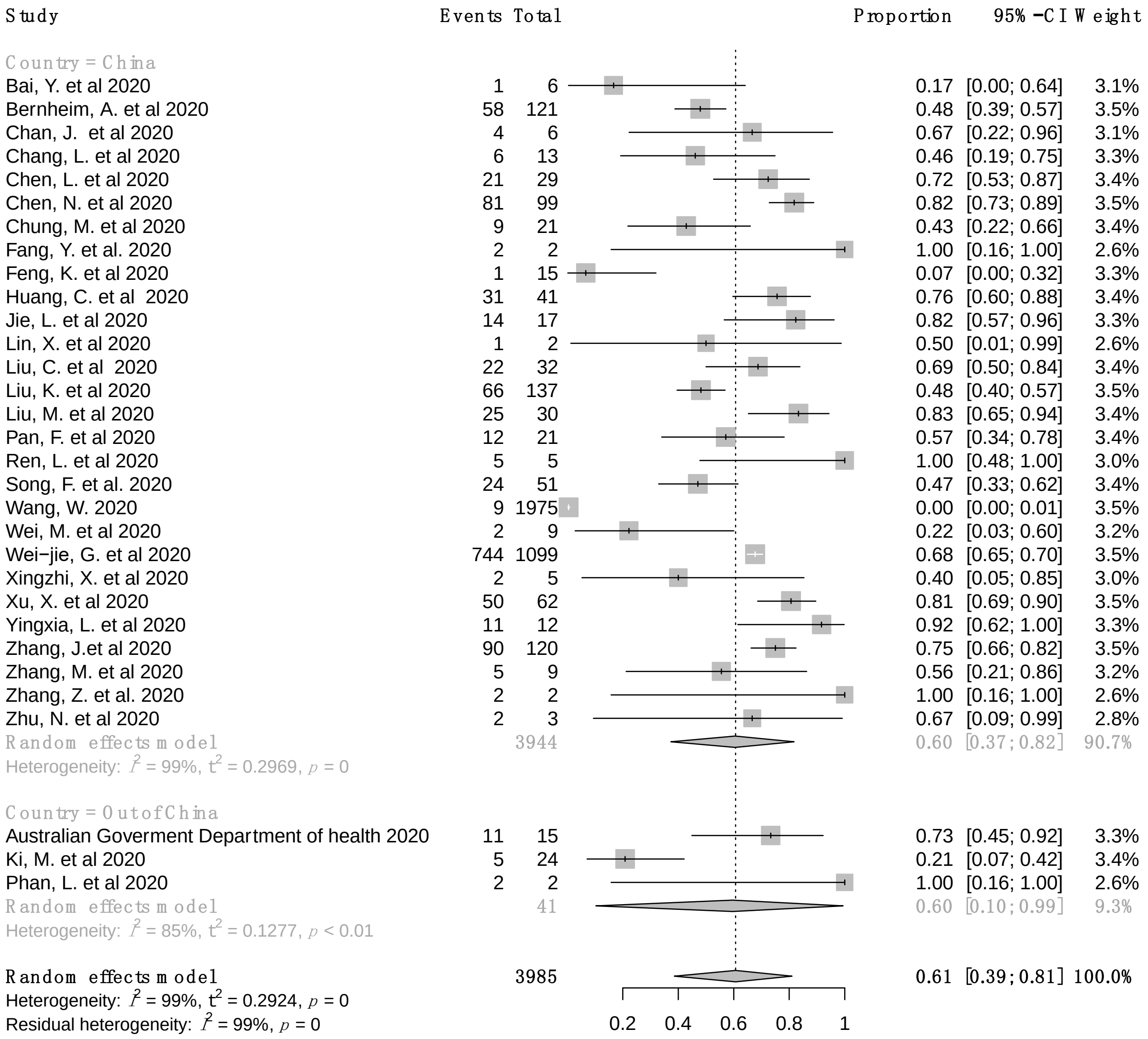

Figure S2. Meta-analysis of the incidence of cough among the selected studies.

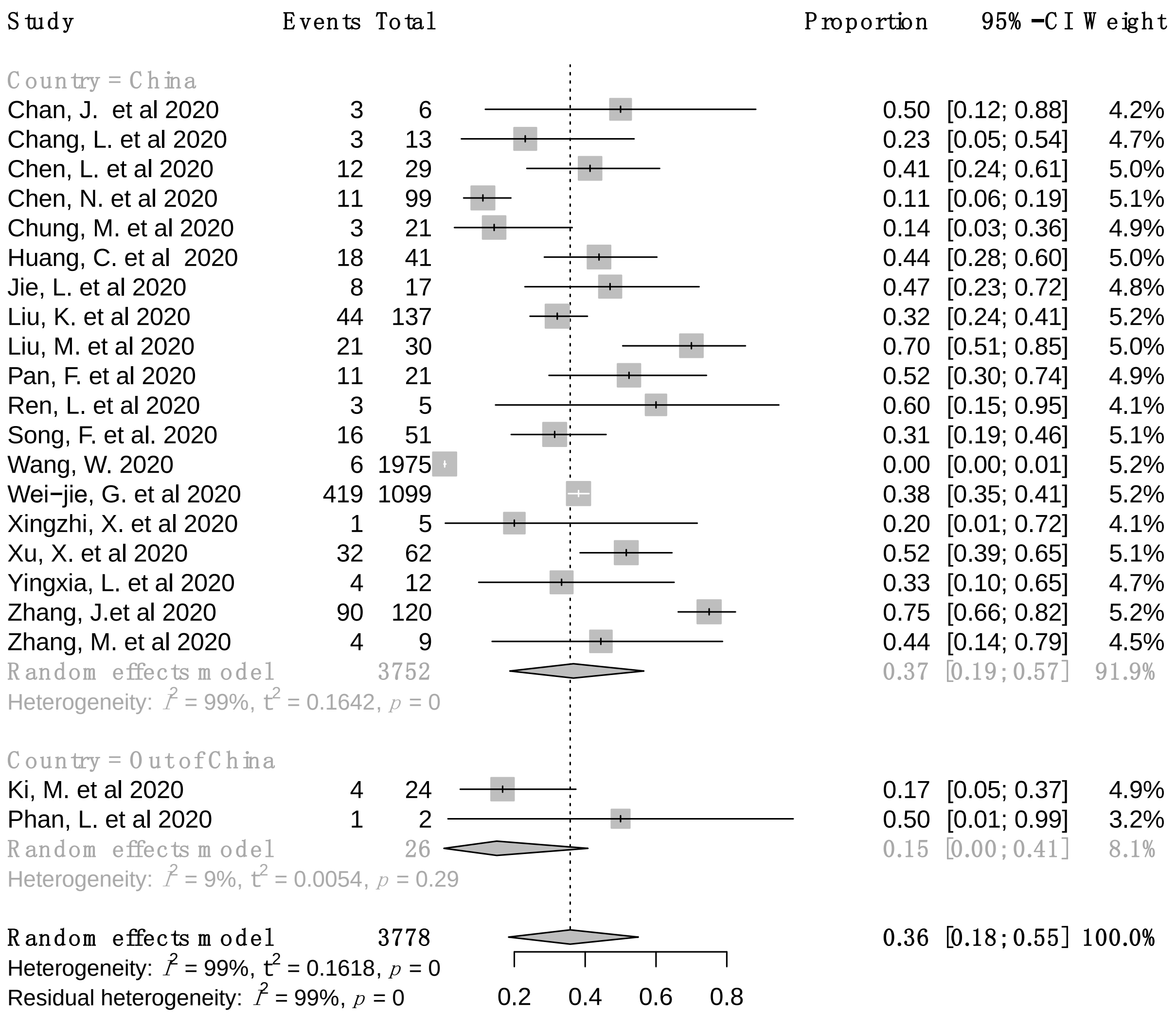

Figure S3. Meta-analysis of the incidence of muscle pain or fatigue among the selected studies.

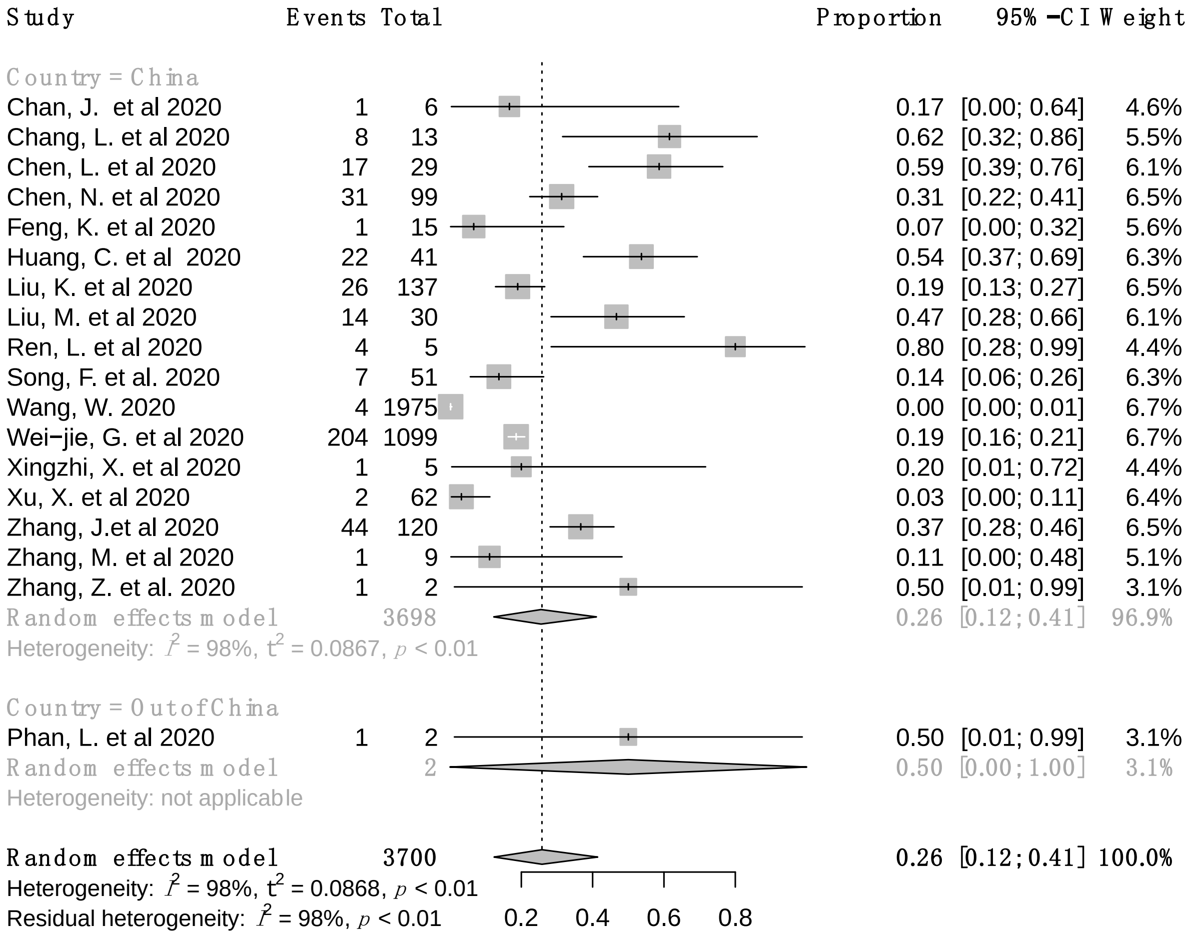

Figure S4. Meta-analysis of the incidence of dyspnea among the selected studies.

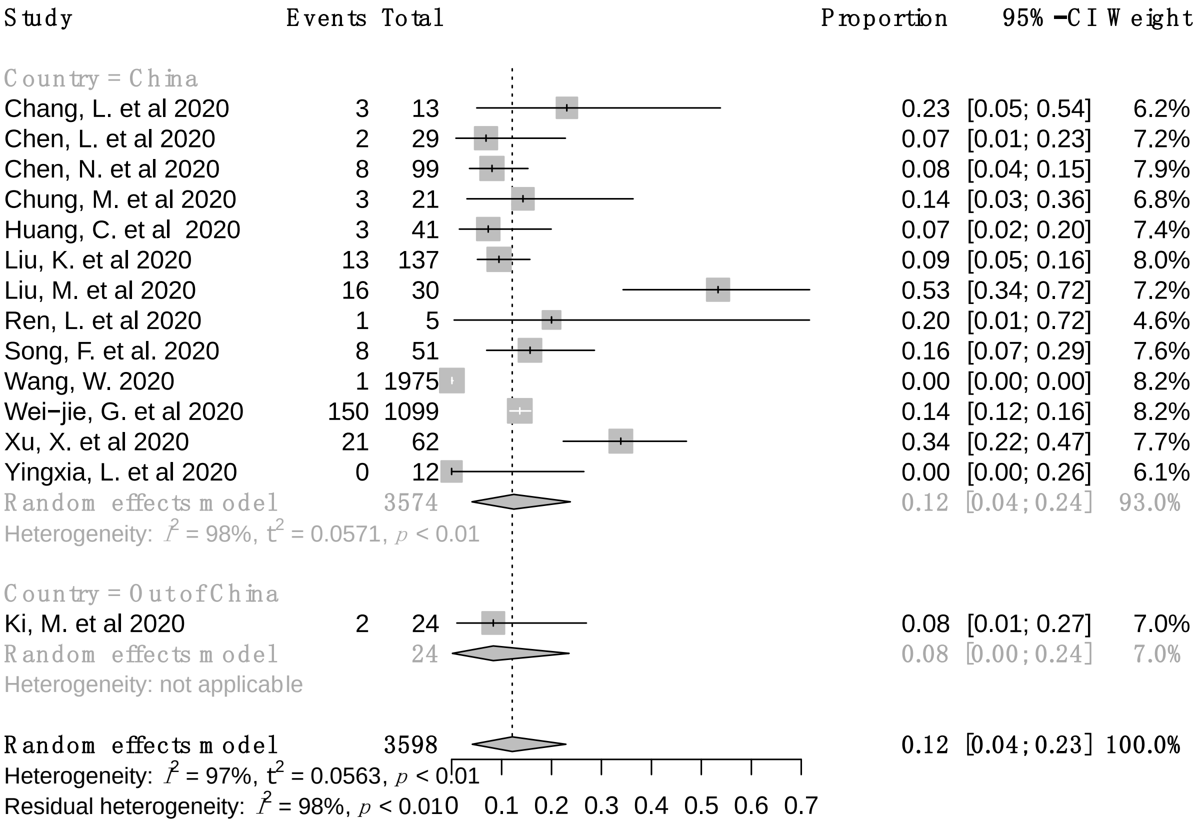

Figure S5. Meta-analysis of the incidence of headache among the selected studies.

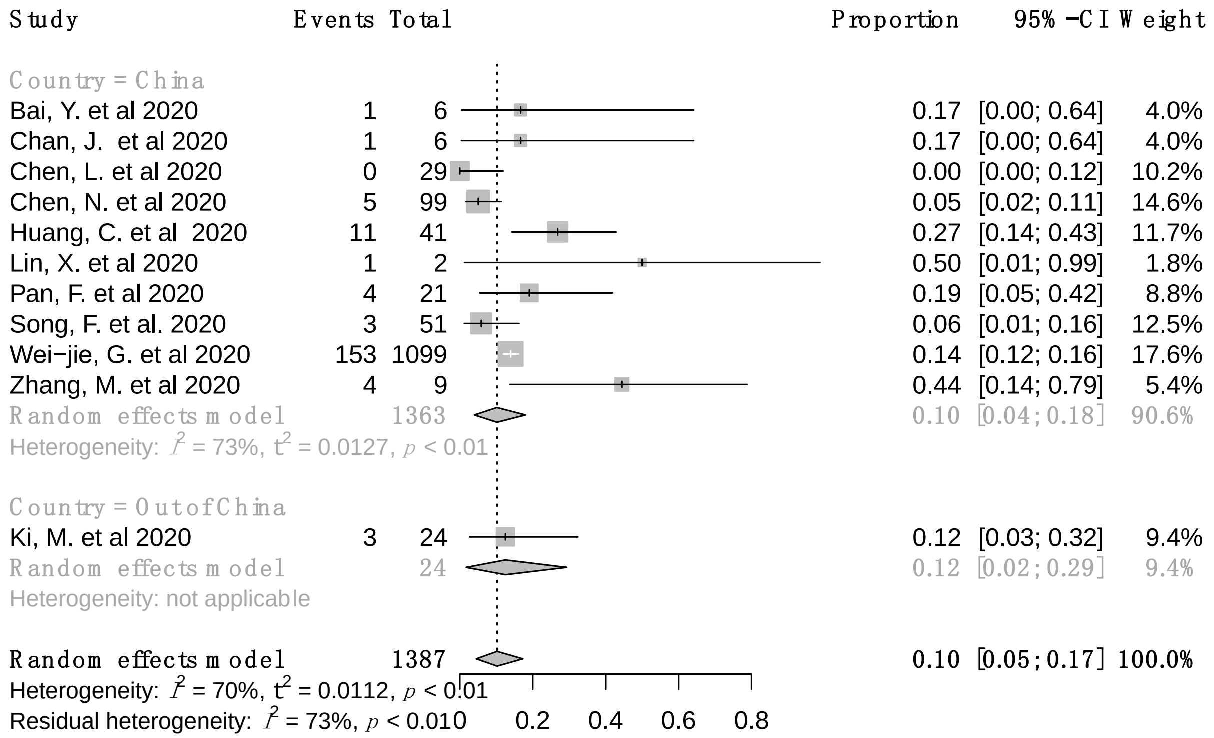

Figure S6. Meta-analysis of the incidence of sore throat among the selected studies.

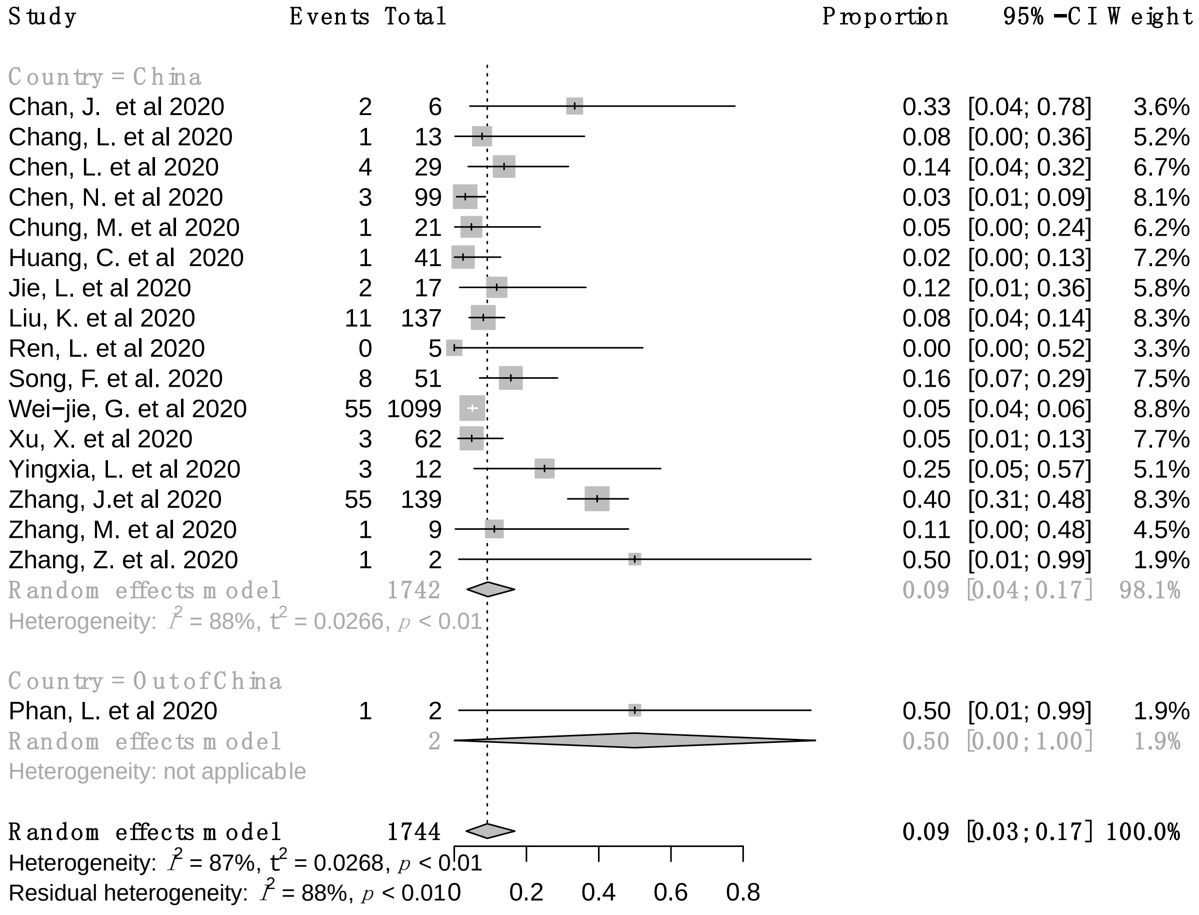

Figure S7. Meta-analysis of the incidence of gastrointestinal disorders among the selected studies.

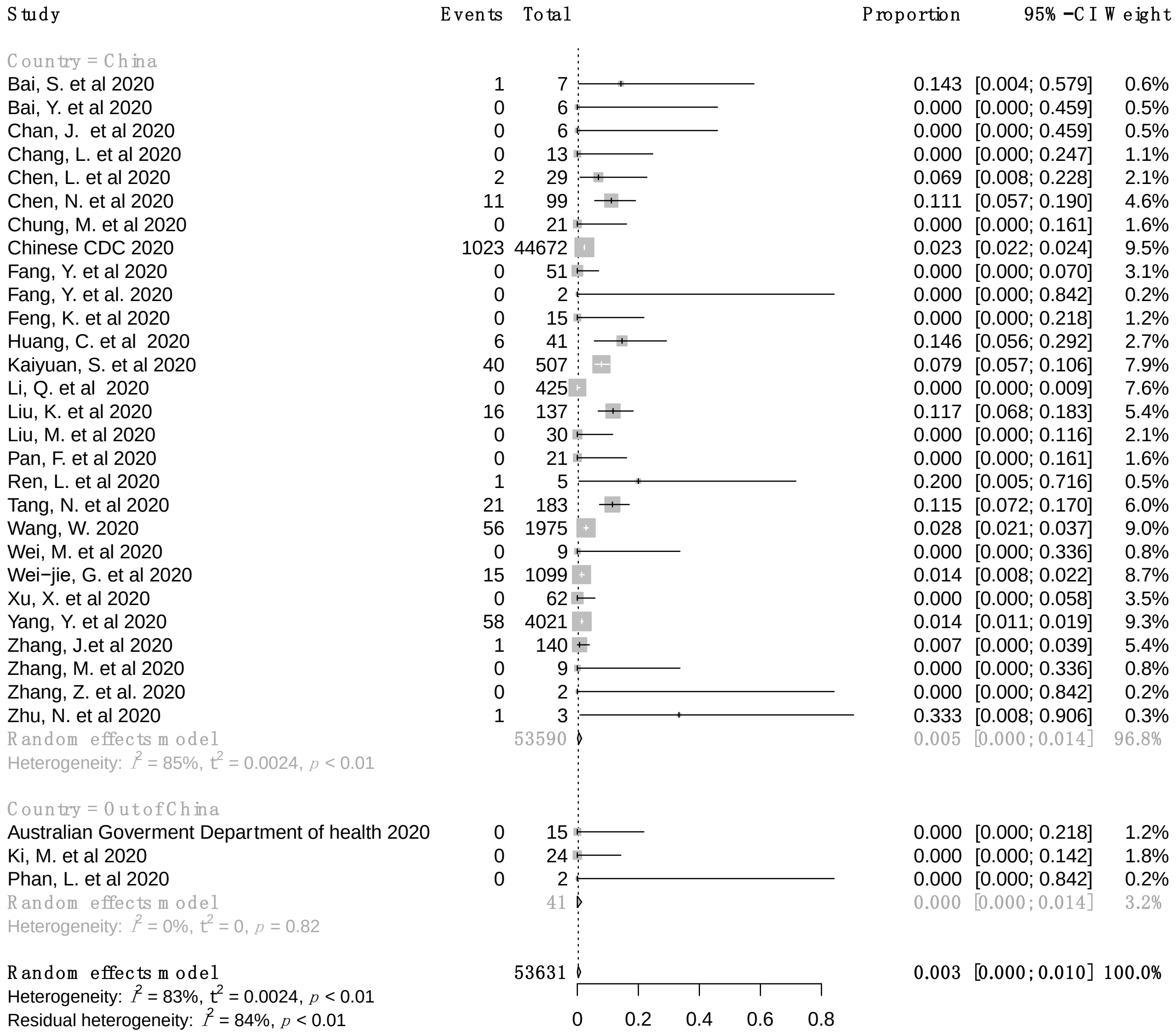

Figure S8. Meta-analysis of mortality among the selected studies.

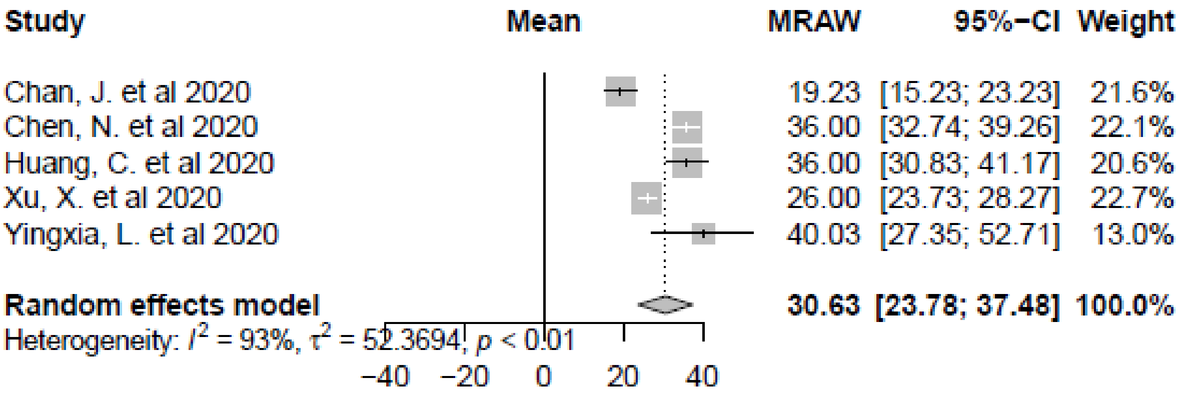

Figure S9. Meta-analysis of the serum levels of AST among the selected studies. **Note:** Analysis based on 220 patients.

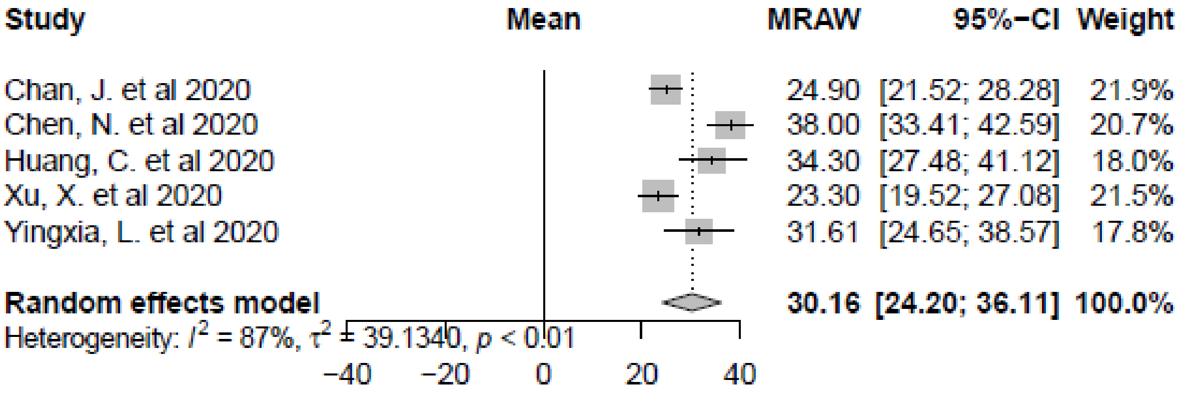

Figure S10. Meta-analysis of the serum levels of ALT among the selected studies. **Note:** Analysis based on 220 patients.

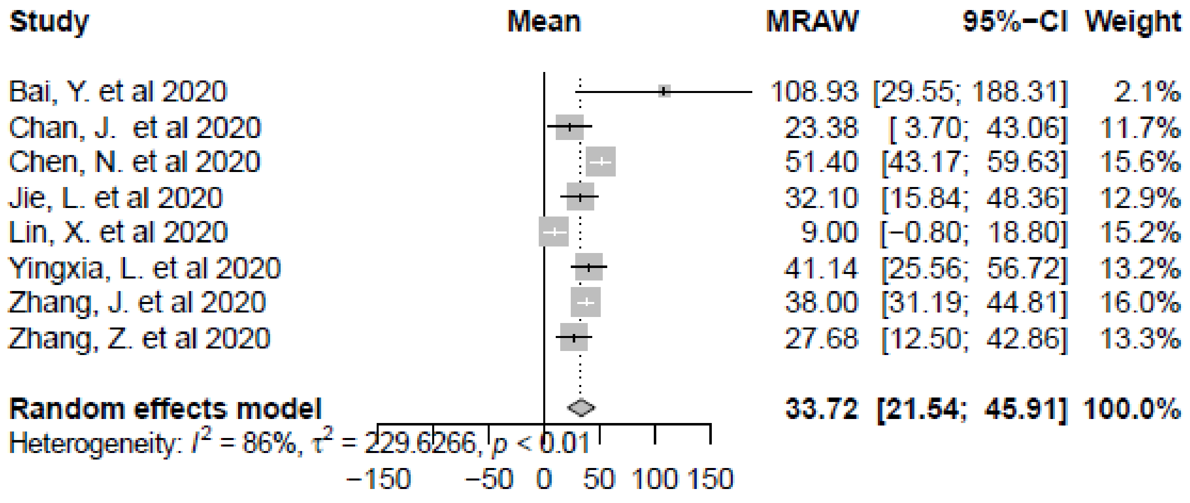

Figure S11. Meta-analysis of the serum levels of C-reactive protein among the selected studies. **Note:** Analysis based on 284 patients; Sensitivity analysis performed withdrawing Lin et al (high variance results based on 2 patients) with following results: MRAW = 38.15 (95%CI 29.36-46.95, I^2 = 64%).

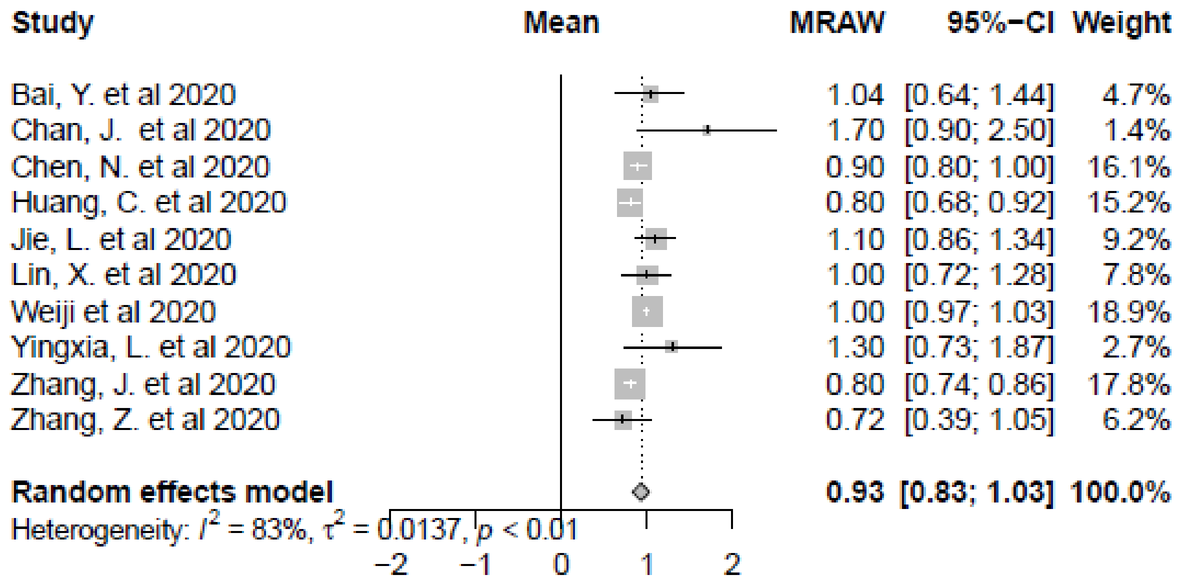

Figure S12. Meta-analysis of the lymphocyte measurement among the selected studies. **Note:** Analysis based on 1424 patients.

References

6 Chan, J.F.-W.; Yuan, S.; Kok, K.-H.; To, K.K.; Chu, H.; Yang, J.; Xing, F.; Liu, J.; Yip, C. C.-Y.; Poon, R. W.-S.; et al. A familial cluster of pneumonia associated with the 2019 novel coronavirus indicating person-to-person transmission: A study of a family cluster. *Lancet* **2020**, *395*, 514–523

16. Chen, L.; Liu, H.; Liu, W.; Liu, J.; Liu, K.; Shang, J.; Deng, Y.; Wei, S. Analysis of clinical features of 29 patients with 2019 novel coronavirus pneumonia. *Zhonghua Jie He He Hu Xi Za Zhi* **2020**, *43*, 203–208.

17. Feng, K.; Yun, Y.; Wang, X.; Yang, G.D.; Zheng, Y.J.; Lin, C.M.; Wang, L. Analysis of CT features of 15 children with 2019 novel coronavirus infection. *Zhonghua Er Ke Za Zhi* **2020**, *58*, E007.

18. Tang, N.; Li, D.; Wang, X.; Sun, Z. Abnormal coagulation parameters are associated with poor prognosis in patients with novel coronavirus pneumonia. *J. Thromb. Haemost*. **2020**, doi:10.1111/jth.14768.

19. Pan, Y.; Guan, H.; Zhou, S.; Wang, Y.; Li, Q.; Zhu, T.; Hu, Q; Xia, L. Initial CT findings and temporal changes in patients with the novel coronavirus pneumonia (2019-nCoV): A study of 63 patients in Wuhan, China. *Eur. Radiol*. Available on: https://link.springer.com/content/pdf/10.1007/s00330-020-06731-x.pdf (Accessed 25 March 2020)

20. Hu, J.; He, G.; Liu, T.; Xiao, J.P.; Rong, Z.H.; Guo, L.C.; Zeng, W.; Zhu, Z.; Gond, D. Yin, L.; et al. Risk assessment of exported risk of novel coronavirus pneumonia from Hubei Province. *Zhonghua Yu Fang Yi Xue Za Zhi* **2020**, *54*, E017.

21. Novel CPERE. The epidemiological characteristics of an outbreak of 2019 novel coronavirus diseases (COVID-19) in China. *Zhonghua Liu Xing Bing Xue Za Zhi* **2020**, *41*, 145–151.

22. Sun, K.; Chen, J.; Viboud, C. Early epidemiological analysis of the coronavirus disease 2019 outbreak based on crowdsourced data: A population-level observational study. *Lancet Digital Health*. **2020**, doi:10.1016/S2589-7500(20)30026-1.

23. Liu, Y.; Yang, Y.; Zhang, C.; Huang, F.; Wang, F.; Yuan, J.; Wang, Z.; Li, J.; Feng, C.; Zhang, Z.; et al. Clinical and biochemical indexes from SARS-CoV-2 infected patients linked to viral loads and lung injury. *Sci. China Life Sci*. **2020**, *63*, 364–374.

24. Robles, A.; San Gil, A.; Pascual, V.; Calbo, E.; Viladot, E.; Benet, S.; Bienvenido, B; Cuchi, E.; Torrer, J.; Canales, L.; et al. Viral vs bacterial community-acquired pneumonia: Radiologic features. *Eur. Respir. J*. **2011**, *38*, 2507.

25. Bernheim, A.; Mei, X.; Huang, M.; Yang, Y.; Fayad, Z.A.; Zhang, N.; Dias, K.; Lin, B.; Zhu, X.; Li, K.; et al. Chest CT findings in coronavirus disease-19 (COVID-19): Relationship to duration of infection. *Radiology* **2020**, *200463*, doi:10.1148/radiol.2020200463.

32. Team C-NIRS. COVID-19, Australia: Epidemiology Report 3 (Reporting week ending 19:00 AEDT 15 February 2020). *Commun. Dis. Intell. (2018)* **2020**, *44*, doi:10.33321/cdi.2020.44.21.

33. Bai, S.; Wang, J.; Zhou, Y.; Yu, D.S.; Gao, X.M.; Li, L.L.; Yang, F.. Analysis of the first cluster of cases in a family of novel coronavirus pneumonia in Gansu Province. *Zhonghua Yu Fang Yi Xue Za Zhi* **2020**, *54*, E005.

34. Bai, Y.; Yao, L.; Wei, T.; Tian, F.; Jin, D.Y.; Chen, L.; Yang, M. Presumed asymptomatic carrier transmission of COVID-19. *JAMA* **2020**, doi:10.1001/jama.2020.2565.

35. Bastola, A.; Sah, R.; Rodriguez-Morales, A.J.; Lal, B.; Jha, R.; Ojha, H.; Shrestha, B.; Chu, D.; Poon, L.; Costello, A.; et al. The first 2019 novel coronavirus case in Nepal. *Lancet Infect. Dis*. **2020**, *20*, 279–280.

36. Cai, J.; Wang, X.; Ge, Y.; Xia, A.; Chang, H.; Tian, H.; Zhu, Y.; Wang, Q.; Zeng, J. First case of 2019 novel coronavirus infection in children in Shanghai. *Zhonghua Er Ke Za Zhi* **2020**, *58*, E002.

37. Chang, D.; Lin, M.; Wei, L.; Xie, L.; Zhu, G.; Dela Cruz, C.S.; Sharma, L. Epidemiologic and clinical characteristics of novel coronavirus infections involving 13 patients outside Wuhan, China. *JAMA* **2020**, doi:10.1001/jama.2020.1623.

38. Chen, F.; Liu, Z.; Zhang, F.; Xiong, R.H.; Chen, Y.; Cheng, X.F.; Wang, W.; Ren, J. First case of severe childhood novel coronavirus pneumonia in China. *Zhonghua Er Ke Za Zhi* **2020**, 58, E005.

39. Chen, N.; Zhou, M.; Dong, X.; Qu, J.; Gong, F.; Han, Y.; Qiu, Y.; Wang, J.; Liu, Y.; Wei, Y.; et al. Epidemiological and clinical characteristics of 99 cases of 2019 novel coronavirus pneumonia in Wuhan, China: A descriptive study. *Lancet* **2020**, *395*, 507–513.

40. Chung, M.; Bernheim, A.; Mei, X.; Zhang, N.; Huang, M.; Zeng, X.; Cui, J.; Yang, Y.; Fayad, Z.; Jacobi, A.; et al. CT imaging features of 2019 novel coronavirus (2019-nCoV). *Radiology* **2020**, *295*, 202–207.

41. Duan, Y.-N.; Qin, J. Pre-and posttreatment chest CT findings: 2019 novel coronavirus (2019-nCoV) pneumonia. *Radiology* **2020**, *295*, 21.
42. Fang, X.; Zhao, M.; Li, S.; Yang, L.; Wu, B. Changes of CT Findings in a 2019 Novel Coronavirus (2019-nCoV) pneumonia patient. *QJM*. **2020**, doi:10.1093/qjmed/hcaa038.
43. Fang, Y.; Zhang, H.; Xie, J.; Lin, M.; Ying, L.; Pang, P.; Ji, W. Sensitivity of chest CT for COVID-19: Comparison to RT-PCR. *Radiology* **2020**, *200432*, doi:10.1148/radiol.2020200432.
44. Fang, Y.; Zhang, H.; Xu, Y.; Xie, J.; Pang, P.; Ji, W. CT manifestations of two cases of 2019 novel coronavirus (2019-nCoV) pneumonia. *Radiology* **2020**, *295*, 208–209.
45. Giovanetti, M.; Benvenuto, D.; Angeletti, S.; Ciccozzi, M. The first two cases of 2019-nCoV in Italy: Where they come from? *J. Med. Virol*. **2020**, *92*, 518–521.
46. Hao, W.; Li, M.; Huang, X. First atypical case of 2019 novel coronavirus in Yan'an, China. *Clin. Microbiol. Infect*. **2020**, doi:10.1016/j.cmi.2020.02.011.
47. Holshue, M.L.; DeBolt, C.; Lindquist, S.; Lofy, K.H.; Wiesman, J.; Bruce, H.; Spitters, C.; Ericson, K.; Wilkerson, S.; Tural, A.; et al. First case of 2019 novel coronavirus in the United States. *N. Engl. J. Med*. **2020**, *382*, 929–936.
48. Huang, C.; Wang, Y.; Li, X.; Ren, L.; Zhao, J.; Hu, Y.; Zhang, L.; Fan, G.; Xu, J.; Gu, X.; et al. Clinical features of patients infected with 2019 novel coronavirus in Wuhan, China. *Lancet* **2020**, *395*, 497–506.
49. Huang, P.; Liu, T.; Huang, L.; Liu, H.; Lei, M.; Xu, W.; Hu, X.; Chen, J.; Liu, B. Use of chest CT in combination with negative RT-PCR assay for the 2019 novel coronavirus but high clinical suspicion. *Radiology* **2020**, *200330*, doi:10.1148/radiol.2020200330.
50. Chen, H.; Guo, J.; Wang, C.; Luo, F.; Yu, X.; Zhang, W.; Li, J.; Zhao, D.; Xu, D.; Gong, Q; et al. Clinical characteristics and intrauterine vertical transmission potential of COVID-19 infection in nine pregnant women: A retrospective review of medical records. *Lancet* **2020**, *395*, 809–815.
51. Ki, M. Epidemiologic characteristics of early cases with 2019 novel coronavirus (2019-nCoV) disease in Korea. *Epidemiol. Health* **2020**, *42*, e2020007.
52. Lei, J.; Li, J.; Li, X.; Qi, X. CT imaging of the 2019 novel coronavirus (2019-nCoV) pneumonia. *Radiology* **2020**, *295*, 18.
53. Li, Q.; Guan, X.; Wu, P.; Wang, X.; Zhou, L.; Tong, Y.; Ren, R.; Leung, K.; Eric, H.; et al. Early transmission dynamics in Wuhan, China, of novel coronavirus–infected pneumonia. *N. Engl. J. Med*. **2020**, 10.1056/NEJMoa2001316.
54. Lin, X.; Gong, Z.; Xiao, Z.; Xiong, J.; Fan, B.; Liu, J. Novel coronavirus pneumonia outbreak in 2019: Computed tomographic findings in two cases. *Korean J. Radiol*. **2020**, *21*, 365–368.
55. Liu, C.; Jiang, Z.; Shao, C.; Zhang, H.G.; Yue, H.M.; Chen, Z.H.; Ma, B.; Liu, W.; Huang, H.; Yang, J.; et al. Preliminary study of the relationship between novel coronavirus pneumonia and liver function damage: A multicenter study. *Zhonghua Gan Zang Bing Za Zhi* **2020**, *28*, 148–152.
56. Kui, L.; Fang, Y.-Y.; Deng, Y.; Liu, W.; Wang, M.F.; Ma, J.P.; Xiao, W.; Wang, Y.; Zhong, M.; Li, C.; et al. Clinical characteristics of novel coronavirus cases in tertiary hospitals in Hubei Province. *Chin. Med. J.* **2020**, doi:10.1097/CM9.0000000000000744.
57. Liu, M.; He, P.; Liu, H.; Wang, X.J.; Li, F.J.; Chen, S.; Liu, J.; Li, C. Clinical characteristics of 30 medical workers infected with new coronavirus pneumonia. *Zhonghua Jie He He Hu Xi Za Zhi* **2020**, *43*, 209–214.
58. Liu, P.; Tan, X.-Z. 2019 novel coronavirus (2019-nCoV) pneumonia. *Radiology* **2020**, *295*, 19.
59. Ministry of Health Labour and Welfare- Japan. Novel Coronavirus—Japan (ex-China). 2020. Available onlie: https://www.who.int/csr/don/17-january-2020-novel-coronavirus-japan-ex-china/en/ (accessed on 21 March 2020).
60. Ministry of Public Health (MoPH) T. Novel Coronavirus—Thailand (ex-China). 2020. Available online: https://www.who.int/csr/don/14-january-2020-novel-coronavirus-thailand-ex-china/en/ (accessed on 21 March 2020).
61. Pan, F.; Ye, T.; Sun, P.; Gui, S.; Liang, B.; Li, L.; Zheng, D.; Wang, J.; Hesketh, R.; Yang, L.; et al. Time course of lung changes on chest CT during recovery from 2019 novel coronavirus (COVID-19) pneumonia. *Radiology* **2020**, *200370*, doi:10.1148/radiol.2020200370.

62. Phan, L.T.; Nguyen, T.V.; Luong, Q.C.; Nguyen, T.V.; Nguyen, H.T.; Le, H.Q. Importation and human-to-human transmission of a novel coronavirus in Vietnam. *N. Engl. J. Med*. **2020**, *382*, 872–874.
63. Pongpirul, W.A.; Pongpirul, K.; Ratnarathon, A.C.; Prasithsirikul, W. Journey of a Thai taxi driver and novel coronavirus. *N. Engl. J. Med*. **2020**, *382*, 1067–1068.
64. Ren, L.-L.; Wang, Y.-M.; Wu, Z.-Q.; Xiang, Z.C.; Guo, L.; Xu, T.; Jiang, Y.; Xiong, Y.; Li, Y.; et al. Identification of a novel coronavirus causing severe pneumonia in human: A descriptive study. *Chin. Med. J.* **2020**, doi:10.1097/CM9.0000000000000722.
65. Rothe, C.; Schunk, M.; Sothmann, P.; Bretzel, G.; Froeschl, G.; Wallrauch, C.; Zimmer, T.; Thiel, V.; Janke, C.; et al. Transmission of 2019-nCoV infection from an asymptomatic contact in Germany. *N. Engl. J. Med*. **2020**, *382*, 970–971.
66. Shi, H.; Han, X.; Zheng, C. Evolution of CT manifestations in a patient recovered from 2019 novel coronavirus (2019-nCoV) pneumonia in Wuhan, China *Radiology*. **2020**, *295*, 20.
67. Silverstein, W.K.; Stroud, L.; Cleghorn, G.E.; Leis, J.A. First imported case of 2019 novel coronavirus in Canada, presenting as mild pneumonia. *Lancet* **2020**, *395*, 734.
68. Song, F.; Shi, N.; Shan, F.; Zhang, Z.; Shen, J.; Lu, H.; Ling, Y.; Jiang, Y.; Shi, Y. Emerging coronavirus 2019-nCoV pneumonia. *Radiology* **2020**, *295*, 210–217.
69. Wang, W.; Tang, J.; Wei, F. Updated understanding of the outbreak of 2019 novel coronavirus (2019-nCoV) in Wuhan, China. *J. Med. Virol*. **2020**, 10.1002/jmv.25689.
70. Wei, M.; Yuan, J.; Liu, Y.; Fu, T.; Yu, X.; Zhang, Z.-J. Novel coronavirus infection in hospitalized infants under 1 year of age in China. *JAMA* **2020**, doi:10.1001/jama.2020.2131.
71. Guan, W.-J.; Ni, Z.-Y.; Hu, Y.; Liang, W.-H.; Ou, C.-Q.; He, J.-X.; Liu, L.; Shan, H.; Lei, C.; Hiu, D.; et al. Clinical characteristics of coronavirus disease 2019 in China. *N. Engl. J. Med*. **2020**, doi:10.1056/NEJMoa2002032.
72. Xie, X.; Zhong, Z.; Zhao, W.; Zheng, C.; Wang, F.; Liu, J. Chest CT for typical 2019-nCoV pneumonia: Relationship to negative RT-PCR testing. *Radiology* **2020**, *200343*, doi:10.1148/radiol.2020200343.
73. Xu, X.; Yu, C.; Zhang, L.; Luo, L.; Liu, J. Imaging features of 2019 novel coronavirus pneumonia. *Eur. J. Nucl. Med. Mol. Imaging* **2020**. doi: https://doi.org/10.1007/s00259-020-04720-2
74. Xu, X.-W.; Wu, X.-X.; Jiang, X.-G.; Xu, K.J.; Ying, L.J.; Ma, C.L.; Wang, H.; Zhang, S.; Gao, H.; et al. Clinical findings in a group of patients infected with the 2019 novel coronavirus (SARS-Cov-2) outside of Wuhan, China: Retrospective case series. *BMJ* **2020**, *368*, m606.
75. Zeng, L.; Tao, X.; Yuan, W.; Wang, J.; Liu, X.; Liu, Z. First case of neonate infected with novel coronavirus pneumonia in China. *Zhonghua Er Ke Za Zhi* **2020**, *58*, E009.
76. Zhang, J.j.; Dong, X.; Cao, Y.Y.; Yuan, Y.D.; Yang, Y.B.; Yan, Y.Q.; Akdis, C.; Gao, Y. Clinical characteristics of 140 patients infected by SARS-CoV-2 in Wuhan, China. *Allergy*. **2020**, doi:10.1111/all.14238.
77. Zhang, M.; Wang, X.; Chen, Y.; Zhao, K.L.; Cai, Y.Q.; An, C.L.; Lin, M.; Mu, X. Clinical features of 2019 novel coronavirus pneumonia in the early stage from a fever clinic in Beijing. *Zhonghua Jie He He Hu Xi Za Zhi* **2020**, *43*, E013.
78. Zhang, Y.; Lin, D.; Xiao, M.; Wang, J.C.; Wei, Y.; Lei, Z.X.; Zeng, Z.; Li, L.; Li, H.; Xiang, W. 2019-novel coronavirus infection in a three-month-old baby. *Zhonghua Er Ke Za Zhi* **2020**, *58*, E006.
79. Zhang, Z.; Li, X.; Zhang, W.; Shi, Z.-L.; Zheng, Z.; Wang, T. Clinical features and treatment of 2019-nCoV pneumonia patients in Wuhan: Report of a couple cases. *Virol. Sin*. **2020**, 1–7. Doi: https://doi.org/10.1007/s12250-020-00203-8
80. Park, W.B.; Kwon, N.-J.; Choi, S.-J.; Kang, C.K.; Choe, P.G.; Kim, J.Y.; Yun, J.; Lee, G.; Seong, M.; Kim, N.; et al. Virus isolation from the first patient with SARS-CoV-2 in Korea. *J. Korean Med. Sci*. **2019**, *35*, e84.
81. Lim, J.; Jeon, S.; Shin, H.-Y.; Kim, M.J.; Seong, Y.M.; Lee, W.J.; Choe, K.; Kang, Y.; Lee, B.; Park, S. Case of the index patient who caused tertiary transmission of COVID-19 infection in Korea: The application of lopinavir/ritonavir for the treatment of COVID-19 infected pneumonia monitored by quantitative RT-PCR. *J. Korean Med. Sci*. **2020**, *35*, e79.

82. Kim, J.Y.; Choe, P.G.; Oh, Y.; Oh, K.J.; Kim, J.; Park, S.J.; Na, H.; Oh, M. The first case of 2019 novel coronavirus pneumonia imported into Korea from Wuhan, China: Implication for infection prevention and control measures. *J. Korean Med. Sci*. **2020**, *35*, e61.
83. Yoo, J.-H.; Hong, S-T. The outbreak cases with the novel coronavirus suggest upgraded quarantine and isolation in Korea. *J. Korean Med. Sci*. **2020**, *35*, e62.
84. Kim, J.Y.; Ko, J.-H.; Kim, Y.; Kim, Y.J.; Kim, J.M.; Chung, Y.S.; Kim, H.; Han, M.; Kin, S.; Chin, B. Viral load kinetics of SARS-CoV-2 infection in first two patients in Korea. *J. Korean Med. Sci*. **2019**, *35*, e86.
85. Team C-NIRS. SARS-CoV-2 acute respiratory disease, Australia: Epidemiology Report 1 (Reporting week 26 January-1 February 2020). *Commun. Dis. Intell. (2018)* **2020**, *44*, doi:10.33321/cdi.2020.44.13.
86. Team C-NIRS. SARS-CoV-2 acute respiratory disease, Australia: Epidemiology Report 1 (Reporting week 1 February-8 February 2020). *Commun. Dis. Intell. (2018)* **2020**, *44*. Doi: https://doi.org/10.33321/cdi.2020.44.14
87. Wang, Z.; Chen, X.; Lu, Y.; Chen, F.; Zhang, W. Clinical characteristics and therapeutic procedure for four cases with 2019 novel coronavirus pneumonia receiving combined Chinese and Western medicine treatment. *BioSci. Trends* **2020**, *14*, 64–68.
88. Chen, L.; Liu, W.; Zhang, Q.; Xu, K.; Ye, G.; Wu, W.; Sun, Z.; Liu, F.; Wu, K.; Zhong, B.; et al. RNA based mNGS approach identifies a novel human coronavirus from two individual pneumonia cases in 2019 Wuhan outbreak. *Emerg. Microbes Infect*. **2020**, *9*, 313–319.
89. Bajema, K.L.; Oster, A.M.; McGovern, O.L.; Lindstrom, S.; Stenger, M.R.; Anderson, T.C.; Isenhour, C.; Clarke, K.; Evans, M.; Chu, V.; et al. Persons evaluated for 2019 novel coronavirus—United States, January 2020. *MMWR. Morb. Mortal Wkly. Rep*. **2020**, *69*, 166–170.
90. Yang, Y.; Qingbin, L.; Mingjin, L.; Wang, Y.; Zhang, A.; Jalali, N.; Dean, N.; Longini, I.; Halloran, E.; Xu, B.; et al. Epidemiological and clinical features of the 2019 novel coronavirus outbreak in China. *medRxiv* **2020**, doi: 10.1101/2020.02.10.20021675. Preprint article.
91. Zhu, N.; Zhang, D.; Wang, W.; Li, X.; Yang, B.; Song, J.; Zhao, X.; Huang, B.; Shi, W.; Lu, R.; et al. A novel coronavirus from patients with pneumonia in China, 2019. *N. Engl. J. Med*. **2020**, *382*, 727–733.
92. Han, W.; Quan, B.; Guo, Y.; Zhang, J.; Lu, Y.; Feng, G.; Fang, F.; Wu, Q.; Cheng, L.; Chen, Q.; et al. The course of clinical diagnosis and treatment of a case infected with coronavirus disease 2019. *J. Med. Virol*. **2020**, *92*, 461–463.
93. Wang, D.; Hu, B.; Hu, C.; Zhu, F.; Liu, X.; Zhang, J. Wang, J.; Wang, B.; Xiang, H.; Cheng, Z.; Xiong, Y.; et al. Clinical characteristics of 138 hospitalized patients with 2019 novel coronavirus—Infected pneumonia in Wuhan, China. *JAMA* **2020**, *323*, 1061–1069.
94. Jei, L.; Li, S.; Cai, Y.; Liu, Q.; Li, X.; Zeng, Z.; Chu, Y.; Zhu, F.; Zeng, F. Epidemiological and clinical characteristics of 17 hospitalized patients with 2019 novel coronavirus infectious outside Wuhan, China. *medRxiv* **2020**, doi:10.1101/2020.02.11.20022053.
